# Supplementary material for: Study on the Migration Behaviors of Magnesium Oxysulfate Nano-Whiskers in Polypropylene Composites with Surface Modification
Source: Materials (Basel). 2023 Aug 29;16(17):5899. doi: 10.3390/ma16175899 (PMC10488912; doi:10.3390/ma16175899)
Supplement: Supplementary file 1 [file materials-16-05899-s001.zip › materials-2548924-supplementary.pdf]

Table S1. Inorganic filler surface EDS analysis result according to surface modification conditions.

Sample	C (wt%)	O (wt%)	Mg (wt%)	S (wt%)
**Whisker**	16.2	59.03	21.05	3.72
**L-5**	47.81	42.88	7.93	1.38
**B-10**	13.46	55.74	24.11	4.62
**B-20**	28.77	51.51	15.9	3.82
**B-30**	34.43	49.76	13.42	2.39
**BM-1**	13.46	57.95	23.96	4.62
**BM-2**	10.65	60.2	24.48	4.66
**BM-4**	29.89	50.79	16.11	3.21

Table S2. XPS analysis results of inorganic filler surface according to surface modification conditions.

Sample	Mg1s (%)	O1s (%)	C1s (%)	S2p (%)	Na1s (%)
**Whisker**	24.55	56.2	14.9	4.35	0
**L-5**	16.63	49.02	31.21	3.14	0
**B-10**	14.87	48.28	29.71	5.19	1.96
**B-20**	12.72	47.17	32.51	5.34	2.27
**B-30**	11.03	42.49	38.51	5.87	2.1
**BM-1**	9.11	42.68	39.04	6.04	3.13
**BM-2**	11.82	45.04	36.86	4.8	1.48
**BM-4**	6.95	38.86	44.74	5.44	4.02

Table S3. Surface EDS analysis results of polypropylene composites during thermally accelerated aging for 0, 24, 48, and 72 h.

Sample	Element	wt%			
		0h	24h	48h	72h
**Whisker**	C	92.17	91.55	90.53	88.28
	O	5.37	5.62	6.31	7.96
	Mg	1.94	2.57	2.56	3.05
	S	0.52	0.26	0.61	0.71
**L-5**	C	97.53	94.47	92.84	95.28
	O	1.78	3.37	5.12	3.01
	Mg	0.54	1.75	1.7	1.27
	S	0.15	0.41	0.34	0.45
**B-10**	C	96.57	96.25	97.36	96.28
	O	2.51	2.78	1.78	2.89
	Mg	0.77	0.67	0.64	0.69
	S	0.15	0.3	0.22	0.14
**B-20**	C	96.48	97.03	97.51	96.76
	O	2.55	2.08	2.01	2.32
	Mg	0.71	0.64	0.37	0.71
	S	0.26	0.25	0.11	0.21
**B-30**	C	96.05	96.28	97.1	95.04

	O	2.79	3.21	2.36	3.68
	Mg	0.93	0.42	0.45	1.06
	S	0.24	0.1	0.09	0.22
**BM-1**	C	95.09	93.68	96.78	89.57
	O	3.32	4.16	2.19	8.13
	Mg	1.29	1.69	0.77	1.93
	S	0.3	0.47	0.26	0.36
**BM-2**	C	95.09	96	95.4	92.07
	O	3.32	2.83	3.18	6.07
	Mg	1.29	0.97	1.09	1.49
	S	0.3	0.21	0.3	0.37
**BM-4**	C	97.53	97.74	97.1	97.61
	O	1.78	1.48	2.49	1.98
	Mg	0.54	0.49	0.32	0.33
	S	0.15	0.29	0.1	0.07

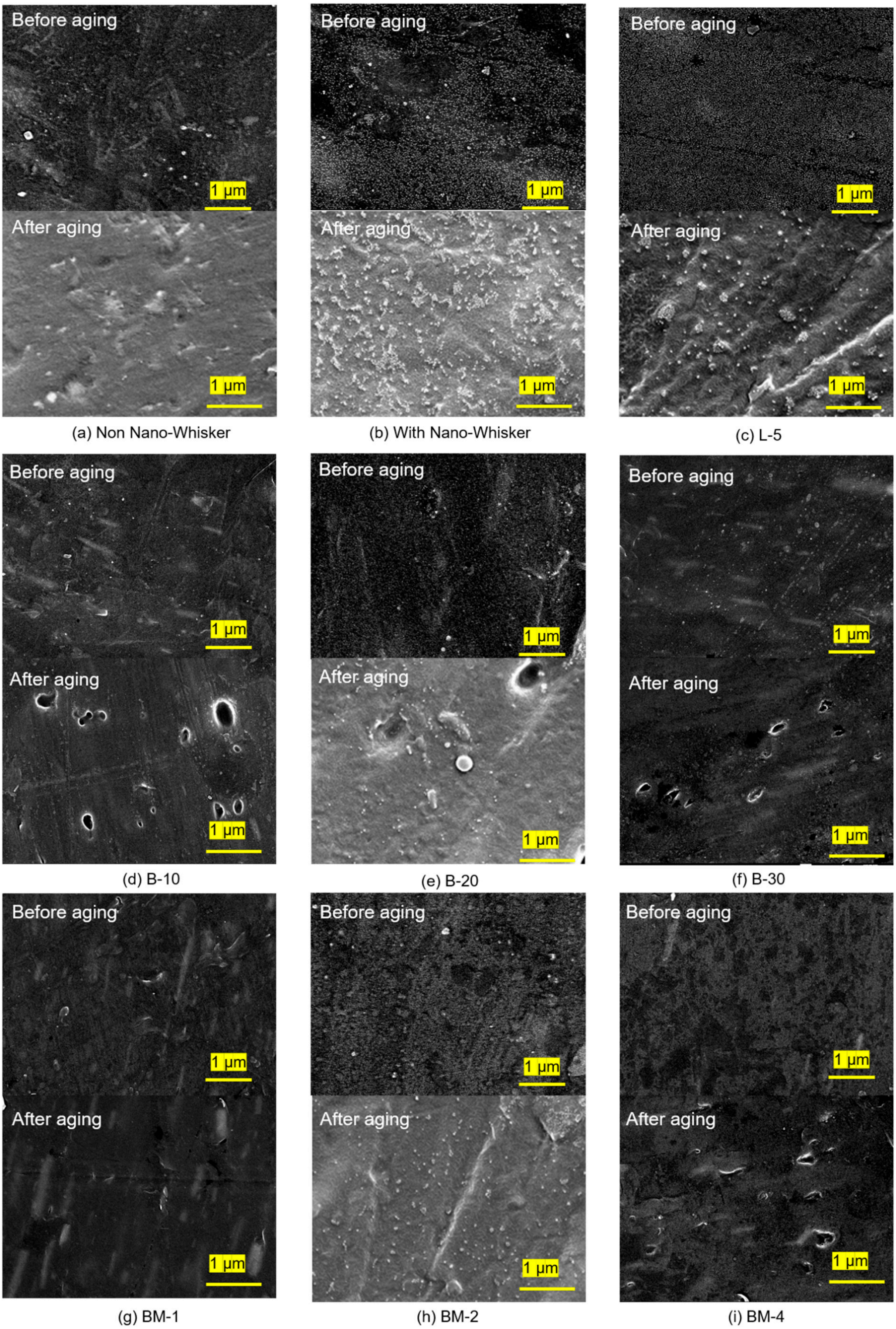

Before aging
After aging
1 µm
(a) Non Nano-Whisker
(b) With Nano-Whisker
(c) L-5
(d) B-10
(e) B-20
(f) B-30
(g) BM-1
(h) BM-2
(i) BM-4

Figure S1. SEM image of the surface of a polypropylene specimen after accelerated thermal aging at 140°C for 72 h. (a) non-Nano-Whisker; (b) with Nano-Whisker; (c) L-5; (d) B-10; (e) B-20; (f) B-30; (g) BM-1; (h) BM-2; (i) BM-4.

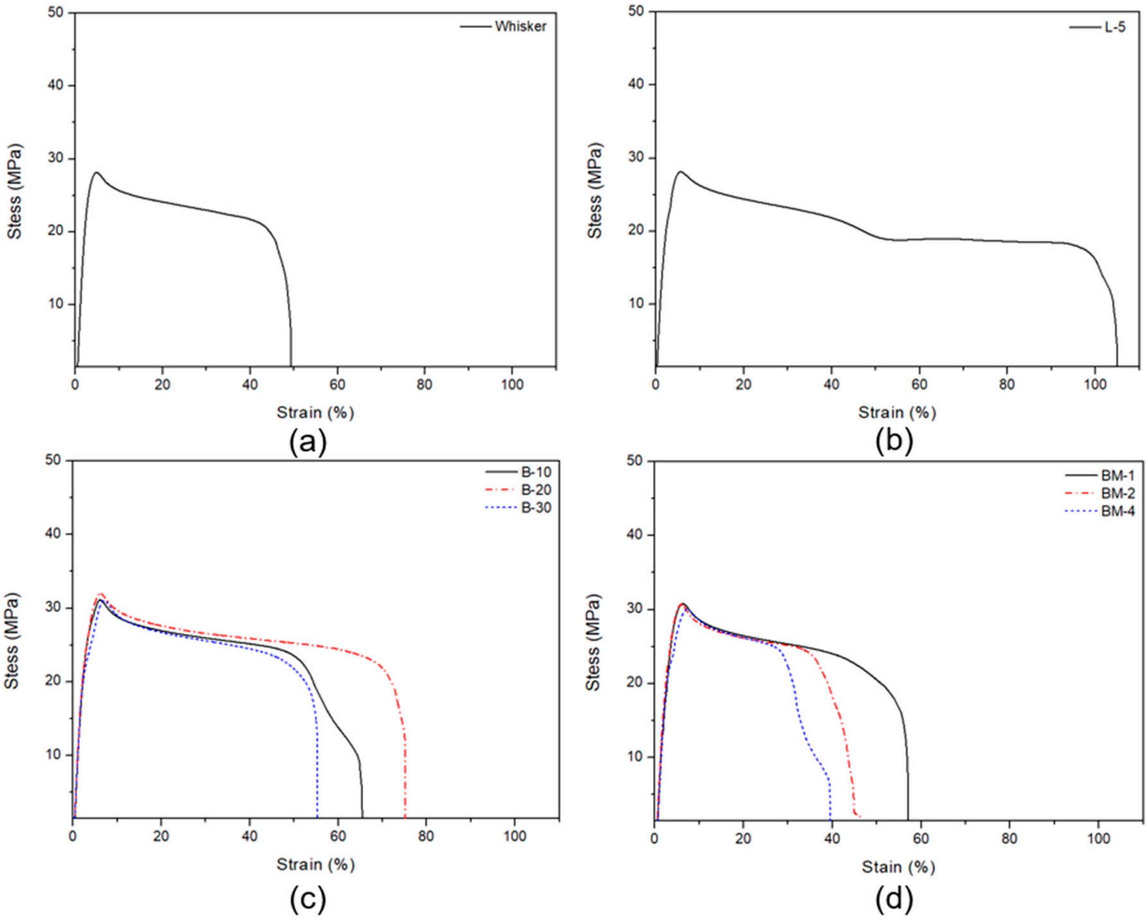

Figure S2. Tensile test S-S curve graphs of polypropylene specimen before accelerated thermal aging. (a) Whisker; (b) L-5; (c) B-10, B-20 and B-30; (d) BM-1, BM-2 and BM-4.

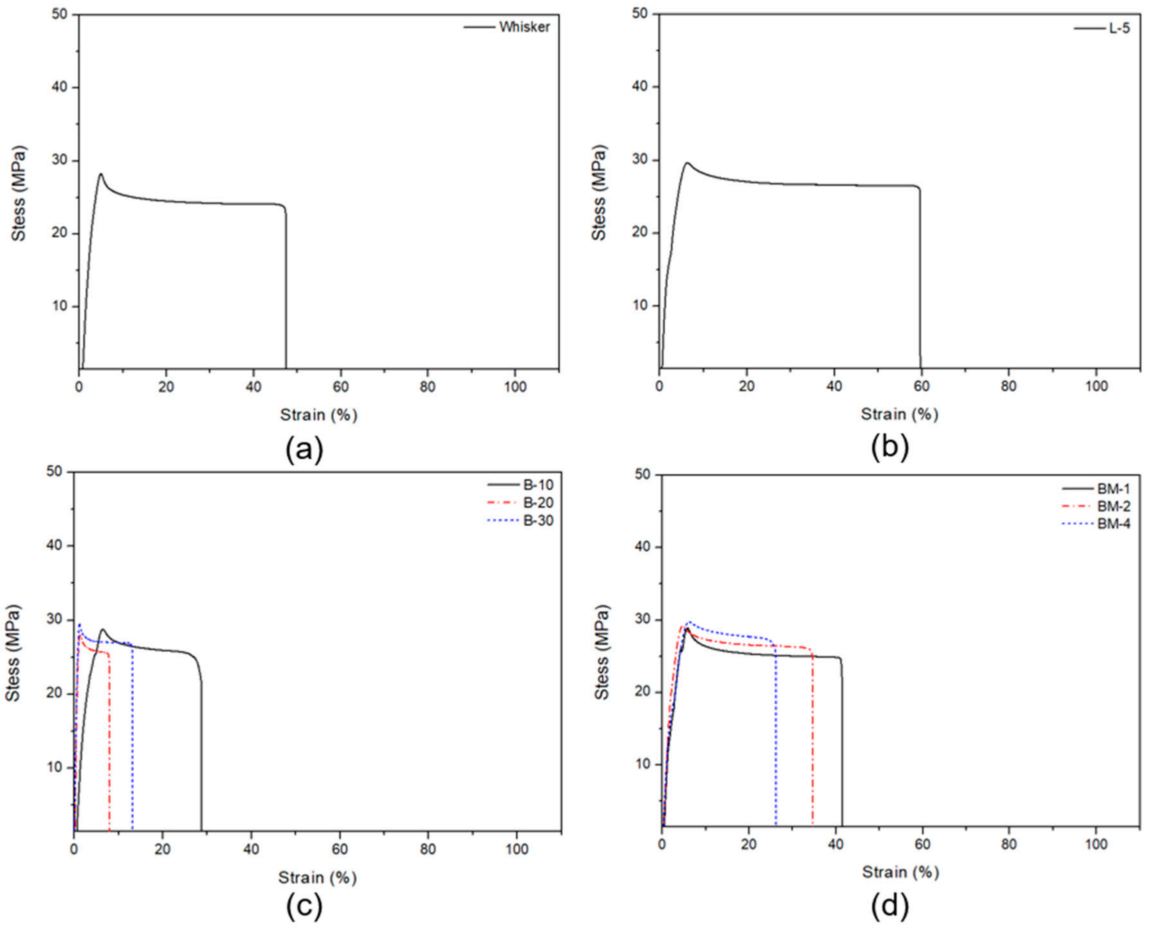

Figure S3. Tensile test S-S curve graphs of polypropylene specimen after accelerated thermal aging. (a) Whisker; (b) L-5; (c) B-10, B-20 and B-30; (d) BM-1, BM-2 and BM-4.
